# Biomarkers of chemotherapy-induced cardiotoxicity: toward precision prevention using extracellular vesicles

**DOI:** 10.3389/fonc.2024.1393930

**Published:** 2024-04-19

**Authors:** Brian B. Silver, Anna Kreutz, Madeleine Weick, Kevin Gerrish, Erik J. Tokar

**Affiliations:** ^1^ Mechanistic Toxicology Branch, Division of Translational Toxicology (DTT), National Institute of Environmental Health Sciences (NIEHS), Durham, NC, United States; ^2^ Molecular Genomics Core, Division of Intramural Research (DIR), National Institute of Environmental Health Sciences (NIEHS), Durham, NC, United States; ^3^ Epigenetics & Stem Cell Biology Laboratory, Division of Intramural Research (DIR), National Institute of Environmental Health Sciences (NIEHS), Durham, NC, United States; ^4^ Inotiv, Durham, NC, United States

**Keywords:** cancer, cardioprotection, cardiotoxicity, chemotherapy, doxorubicin, extracellular vesicles, precision prevention

## Abstract

Detrimental side effects of drugs like doxorubicin, which can cause cardiotoxicity, pose barriers for preventing cancer progression, or treating cancer early through molecular interception. Extracellular vesicles (EVs) are valued for their potential as biomarkers of human health, chemical and molecular carcinogenesis, and therapeutics to treat disease at the cellular level. EVs are released both during normal growth and in response to toxicity and cellular death, playing key roles in cellular communication. Consequently, EVs may hold promise as precision biomarkers and therapeutics to prevent or offset damaging off-target effects of chemotherapeutics. EVs have promise as biomarkers of impending cardiotoxicity induced by chemotherapies and as cardioprotective therapeutic agents. However, EVs can also mediate cardiotoxic cues, depending on the identity and past events of their parent cells. Understanding how EVs mediate signaling is critical toward implementing EVs as therapeutic agents to mitigate cardiotoxic effects of chemotherapies. For example, it remains unclear how mixtures of EV populations from cells exposed to toxins or undergoing different stages of cell death contribute to signaling across cardiac tissues. Here, we present our perspective on the outlook of EVs as future clinical tools to mitigate chemotherapy-induced cardiotoxicity, both as biomarkers of impending cardiotoxicity and as cardioprotective agents. Also, we discuss how heterogeneous mixtures of EVs and transient exposures to toxicants may add complexity to predicting outcomes of exogenously applied EVs. Elucidating how EV cargo and signaling properties change during dynamic cellular events may aid precision prevention of cardiotoxicity in anticancer treatments and development of safer chemotherapeutics.

## Introduction

Rapid, non-invasive strategies for testing and routine monitoring of changes at the cellular level are critical toward understanding chemical and molecular carcinogenesis and preventing symptomatic onset of devastating diseases such as cancer (1, 2). Liquid biopsies, consisting of blood or other bodily fluids, are gaining considerable interest in the scientific community. Biofluids contain a wealth of potential information that could be harnessed toward detecting early cancerous phenotypes (individual or multiple cancers simultaneously) and monitoring response to anticancer therapeutics (3–6). Numerous proteins and nucleic acids, both free-floating and encapsulated within extracellular vesicles (EVs), are released from the cells of internal tissues and are present in extracellular fluids (2, 7, 8).

EVs are a diverse family of membrane-bound particles. EVs were discovered in the early 1980s and first proposed to function as waste transporters for the cell (9). However, coinciding with the finding that EVs contain RNA, the view shifted to consider these particles as potential mediators in cellular communication (10–12). The bi-layered lipid structure of EVs provides a stable means for intercellular transport of a variety of biomolecules, including nucleic acids, both locally and over long distances (13–17). Although a full description of the breadth of cargo identified in EVs would be immense, and is beyond the scope of this article, we point the reader to several excellent reviews and proteomic studies on this topic (18–21).

EVs can participate in cellular communication through fusing with the cellular plasma membrane of target cells and releasing their contents, or through signaling cascades: for example, by binding to receptors on tumor cells to trigger apoptosis (17, 22). EVs display several surface proteins including glycoproteins, tetraspanins, and adhesion molecules that contribute to determining the eventual target and distribution of EVs (17). With their diverse size and composition, EVs play a variety of roles in development and disease. The pleiotropic roles of EVs are well exemplified in the cardiovascular system. Many cells of the cardiovascular system release EVs, including endothelial cells, cardiomyocytes, stem cells, and progenitor cells, both during normal development and in response to disease (23).

EVs may be produced through a variety of mechanisms, including the endocytic pathway (exosomes) (17), budding of the plasma membrane (microvesicles) (24), and as the result of cellular death mechanisms such as apoptosis (apoptotic bodies) (25). These EVs can go on to mediate cardioprotective signaling responses, but EVs can also contribute to cardiotoxicity. Harnessing the cardioprotective properties of EVs and minimizing mechanisms of cardiotoxicity would be of great value for cancer therapies. For instance, many anticancer therapeutics can cause deleterious off-target cardiotoxic effects (26), a classic example being the anthracycline doxorubicin (DOX) (27). In this article, we present our perspective on the potential of EVs as tools to prevent cardiac damage resulting from chemotherapies, both as biomarkers of early cardiotoxicity and as therapeutic cardioprotective agents.

## Extracellular vesicles as biomarkers of cardiac health and cardiotoxicity

The deleterious effects of cardiotoxicity caused by some chemotherapeutic agents are multifaceted and include changes in calcium signaling that cause arrhythmias, cardiac hypertrophy, myocardial remodeling, and cellular death (28). Aberrant cellular death in cardiac tissue is of primary concern as it is a driver of cardiac malfunction and disease (29). Cardiotoxicants often lead ultimately to increased cellular death. For example, DOX can trigger numerous cellular death pathways including apoptosis, ferroptosis, autophagy, necrosis, and pyroptosis (30). EVs are of potential value for detecting tissue-level events because they both represent the current state of a given cell and contain cargo representative of past events. For instance, cells that have been exposed to biochemical or mechanical stimuli such as glucose deprivation or stretch release EVs enriched in receptors for glucose or angiotensin, respectively (31). Overall levels of EVs can be an indicator of cardiovascular disease (24). Developing the ability to isolate and trace EV populations back to specific events, such as toxicity, in their parent tissues is an exciting prospect. Circulating EVs could thus serve as early indicators of cardiac malfunction (32, 33), which could be of value for monitoring cardiac health during chemotherapy.

**Figure 1:**
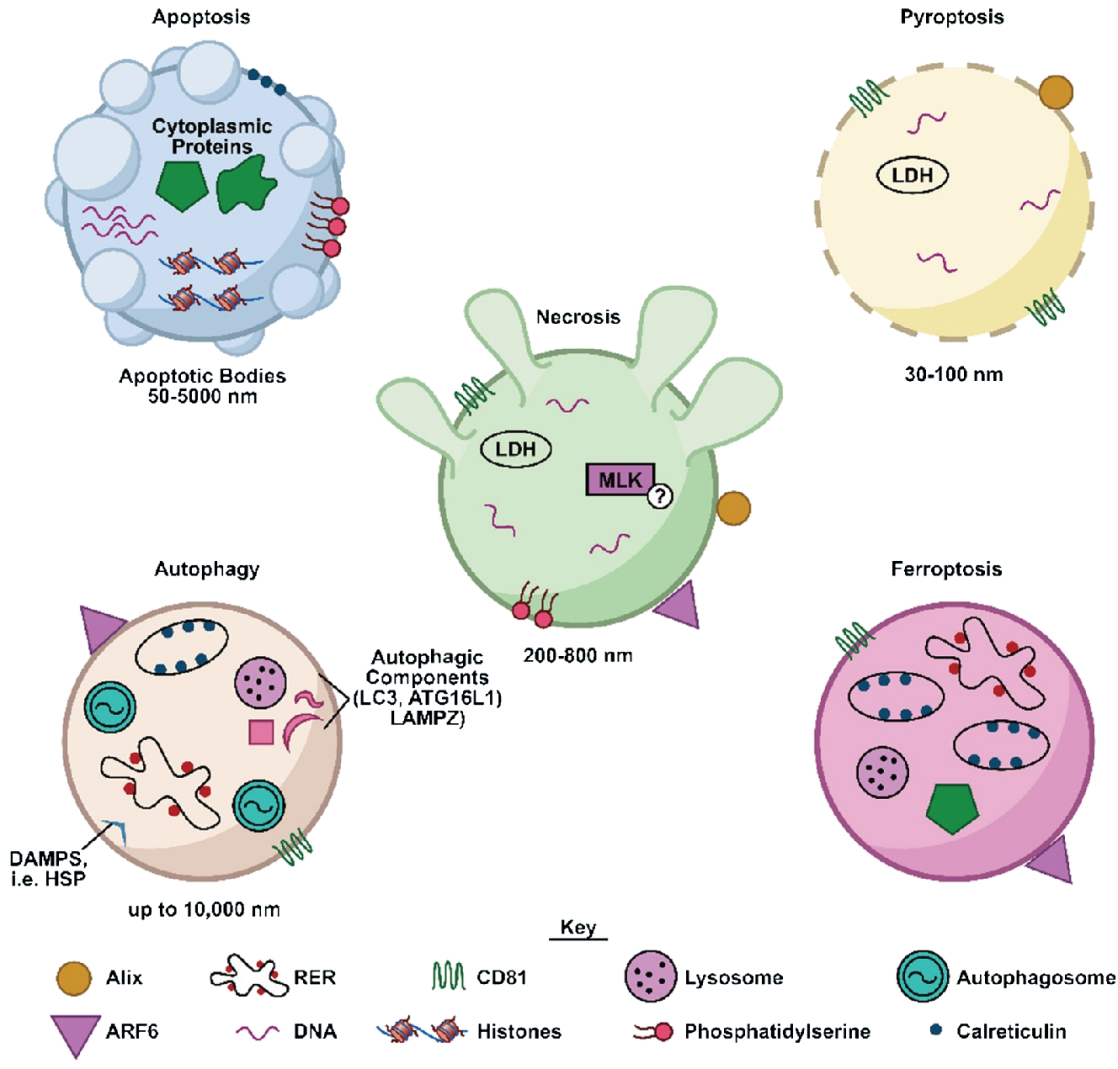
Characteristics of EVs produced in response to different cellular death pathways. Shown is a schematic illustrating characteristics of EVs (cargo, surface proteins) released by cells undergoing several different forms of cellular death. DAMPS, damage-associated molecular patterns; HSP, heat shock proteins; LDH, lactate dehydrogenase; MLK, mixed lineage kinase; RER, rough endoplasmic reticulum.

Several EV properties including size, protein composition, and nucleic acids are altered upon cellular demise, providing a potential means by which to communicate tissue-level events such as cardiotoxicity. Cells undergoing a death pathway have the potential to generate very large (>1000 nm) EVs compared to healthy cells. Isolation and characterization of these large vesicles may provide information about the prevalence and pathways of cellular death initiated in response to a cardiotoxic exposure (**Figure 1**). For instance, apoptotic vesicles can range from 50 -5000 nm (34). Vesicles released from necrotic cells are generally slightly smaller at 200-800 nm (35). EVs of a variety of sizes (30 nm-1000 nm) can be generated in response to several forms of lytic cell death, specifically primary and secondary necrosis and pyroptosis (36). Autophagic processes can generate vesicles (autophagosomes) up to 10 μm in diameter, containing parts of cellular organelles or even intact mitochondria (37).

Further clues as to the origin of an EV may be contained in its associated proteins and cargo. Proteins such as such as CD81, CD63, and CD9 may be indicative of EVs secreted actively via an intracellular pathway (38), whereas vesicles that bud from the plasma membrane during apoptotic or necrotic processes may contain specific cell surface markers (39). For example, both apoptotic and necrotic cells release vesicles presenting phosphatidylserine (35). In addition, EVs expelled from cells undergoing necroptosis, a regulated form of necrosis, carry a key marker of necroptosis, pMLKL (35). Protein cargo can also be significantly altered. For example, a total of 24 EV-associated proteins were found to be differentially regulated in response to apoptosis (40). In addition, EVs generated during apoptosis can encompass fragments of nuclei and thus contain genomic DNA and related proteins such as histones, or stem from the plasma membrane and contain cytoplasmic cargo (34). These vesicles were found to differ from EVs released during autophagy, in that their proteome was depleted of nuclear proteins, and enriched in cytoskeletal and mitochondrial proteins (37).

EV-encapsulated nucleic acids provide both a history of events and can confer downstream effects. For instance, the long noncoding RNA (lncRNA) GAS5 was elevated in atherosclerotic plaques in both patients and humans, and EVs containing lncRNA GAS5 promoted endothelial cell apoptosis (41). EVs commonly contain microRNAs (miRNAs), which play numerous signaling roles and may confer gene silencing through mRNA degradation or repression of protein translation (42). miRNAs involved in the propagation of deleterious signaling pathways can be released in EVs from unhealthy or dying cells. For example, oxidative stress caused release of EV-packaged miR-185-5p, which enhances caspase activity and promotes both apoptosis and necrosis (43). Irradiation of human whole blood samples induced upregulation of EV-containing miR-204-5p, miR-92a-3p, and miR-31-5p, which are involved in pathways regulating apoptosis, proliferation, and immune response (44). Although miRNA transfer is believed to be a primary means through which EVs mediate intercellular signaling, they can also signal via cytokines. For instance, cardiomyocytes expel EVs enriched in TNF-α in response to hypoxia *in vitro*, which can go on to promote further cell death in an autocrine manner (30). Although many forms of cellular death protect tissues by eliminating damaged cells, EVs released from dying cells can be a means for propagating deleterious protein machinery within a stressed tissue. For example, autophagy-dependent ferroptosis induced by oxidative stress was found to promote propagation of mutant KRAS to other cell types via EVs (45). Understanding EV-cargo released by cardiac tissues undergoing cellular death in response to toxic insult may uncover EVs that could serve as biomarkers of early cardiotoxicity.

However, programmed cellular death is not necessarily an isolated outcome that reflects toxicity or poor tissue health. Recently, it has been identified that cells can recover from apoptosis after events including caspase-activation, mitochondrial fragmentation, and DNA damage (46). A separate cell death pathway, ferroptosis, was also found to be reversible (47). Mechanisms through which cells can recover from death pathways may serve to protect valuable cell populations such as cardiomyocytes. However, such processes also drive DNA damage, micronuclei formation, and massive genetic rearrangements, which can lead to cancerous mutations and deleterious phenotypes (46). Not surprisingly, RNA transcription was proposed to be a critical step in apoptosis recovery (46). Thus, cells undergoing recovery from a death pathway might be expected to release different sets of EV-encapsulated cargo than homeostatic cells. An important question is whether these vesicles reflect beneficial or deleterious phenotypes. Knowledge of the differences in EVs released from cells that undergo cellular death versus from cells that recover could aid our ability to use EVs as biomarkers. Using released EVs to identify early cardiotoxic and tumorigenic processes could help predict tissue fate and guide preventative therapeutics.

EVs have already shown promise as biomarkers of DOX-induced cardiotoxicity in mouse models. Specifically, DOX treatment was observed to increase serum EVs that were larger and irregularly shaped, with an increase in protein cargo specific to several key organs including heart and skeletal muscle (48). Notably, these EV-associated protein markers increased earlier and in greater proportions than traditional markers of cardiac disease such as cardiac Troponin-I. In addition to proteins, miRNA within EVs may also reflect DOX-induced toxicity. In dogs undergoing chemotherapy, three serum EV-associated miRNAs were differentially regulated post-DOX treatment (49). Already, such biomarkers have shown potential for clinical utility in humans. Notably, EV-associated miRNA from survivors of acute lymphoblastic leukemia post-DOX chemotherapy were found to be differentially regulated compared to control individuals (50). Further, EV-associated miR-144-3p specifically was correlated with cardiomyopathy. Together, these research efforts suggest that EVs have propensity as biomarkers for cardiotoxicity monitoring. Still, in order for EVs to be successfully implemented as clinical monitoring tools, several challenges must be overcome. Specifically, one limitation is differences in protocols and storage methods for EV isolation across studies, which can alter EV populations and cargo identified (51). Also, the small size and low abundance of some EVs requires increasingly sensitive assays to detect (52). Further development of specific and sensitive methods of EV isolation and detection should bring us closer to identifying EVs that could serve as robust clinical biomarkers. Yet, in addition to using EVs as predictive tools, EVs might themselves be harnessed as cardioprotective agents.

## Harnessing extracellular vesicles as cardioprotective agents

Some major goals of EV-based therapeutics are administering cardioprotective EVs immediately post myocardial infarction (MI) (31) to promote cell survival and developing EV-based strategies for inhibiting cardiotoxicity of cancer drugs. Multiple cell types in the heart including fibroblasts, cardiomyocytes, endothelial cells, and cardiac progenitor cells have been found to release EVs that mediate cellular crosstalk, and play numerous roles in cardiac health including protection from atherosclerosis and modulation of inflammation (31, 53, 54). Due to their plasticity, differentiation capacity, flexibility, and renewal capacity, stem cells have been identified as a promising source of EVs with therapeutic potential. Embryonic stem cell (ESC)-derived EVs have been observed to increase survival, proliferation, neovascularization, and reduce fibrosis in mouse MI models (55). 3D cardiospheres, enriched in stem cells, also release EVs that were shown to be protective in MI models (56). Induced pluripotent stem cell (iPSC)-derived EVs have also been shown to protect against apoptosis following oxidative stress and MI (57). EVs derived from mesenchymal stem cells (MSCs) also possess protective properties. Bone marrow MSC-derived EVs may reduce fibrosis and improve cell function and survival (58). Endometrial MSC-derived EVs may be even more cardioprotective than bone marrow or adipocyte MSCs in some contexts (59). Cardiac stem cells pretreated with EVs produced by MSCs performed better in mouse MI models and showed enhanced proliferation, migration, and vascularization in comparison to untreated counterparts through regulation of numerous miRNAs (60). Many miRNAs have been identified to regulate fibrosis through the TGF-β and NF- κB signaling pathways (61). In addition, excellent reviews have been written summarizing the actions of various miRNAs in additional cardiotoxic processes including apoptosis and inflammation (62, 63).

The cardioprotective properties of EVs may be further exploited to deter the cardiotoxic off-target effects of chemotherapeutics such as DOX via several miRNA-mediated signaling pathways. In mice, systemic delivery of cardiac progenitor cell-derived EVs was found to inhibit cardiotoxicity induced by DOX and trastuzumab, a common breast cancer drug, in a manner dependent on upregulation of miR-146a-5p (64). EVs isolated from bone marrow MSCs attenuated the cardiotoxic effects of DOX in rats through delivery of miRNA-96 (30). In addition, ultrasound-targeted microbubble destruction-assisted EV delivery of miRNA-21 to the heart reduced DOX toxicity in mice (65). EVs have been further implicated in the response and mediation of DOX cardiotoxicity through circular RNA spindle and kinetochore-associated protein 3 (circ-SKA3), miRNA-1303, and Toll-Like Receptor 4 (TLR4) (66). Transfer of lncRNAs such as NEAT1 through EVs was also observed to reduce the cardiotoxic effects of DOX in mice via inhibition of miRNA-221-3p (67). Microvesicles released from apoptotic DOX-treated cells also protected mice from later tumor formation (68). In addition, treatment with ESC-derived EVs decreased inflammation and pyroptosis in response to DOX treatment (69). The breadth of cardioprotective capabilities observed in EVs suggests they may have use as therapeutic tools in the clinic. Yet, unfortunately, not only can cardioprotective properties be transmitted by EVs, but also deleterious phenotypes.

Whether an EV will trigger a cardioprotective or cardiotoxic response depends highly on the identity and history of the cells that released them. For example, although EVs from ESCs appear to promote survival and function of cardiac cells following either DOX exposure or induced MI in mice, EVs isolated from embryonic fibroblasts do not show these cardioprotective benefits (69, 70). Rather, EVs collected from fibroblasts can induce cardiomyocyte hypertrophy (71). It is becoming increasingly apparent that EV-mediated signaling is dependent on the differentiation status, microenvironment, and past events of the cells that released them. These factors can profoundly impact the phenotypes transmitted by released EVs, and their ability to promote cardiac tissue health. For example, EVs derived from cardiomyocytes preconditioned by hypoxia or angiotensin have been observed to promote fibrosis by transferring miRNA-208a to fibroblasts (72). In addition to promoting fibrosis or hypertrophy, EVs can serve additional deleterious roles, including cancer drug-resistance via miRNA transfer (73). Strategies aiming to destroy certain cell populations (such as tumorigenic cells) and preserve or repair other tissues (such as cardiac) require an understanding of the numerous and dichotomous roles that EVs can play.

EVs transmit signals which can trigger the survival or demise of cardiac cells, with cell type and microenvironmental cues impacting their release (33). This opens opportunities for generating therapeutic EVs *in vitro* by guiding the phenotype of the cells from which they are produced. However, the response that will be induced by a given EV type is not necessarily clearly predictable. Intriguingly, for example, prior treatment of cardiac progenitor cells with H_2_O_2_ to induce oxidative stress enhanced the ability of released EVs to attenuate H_2_O_2_-induced apoptosis via miR-21 transfer (23). Successful administration of therapeutic EVs also depends on our ability to target these vesicles within the body. For instance, in mouse models, macrophage engulfment of EVs resulted in sequestration in the liver and spleen, preventing them from reaching their intended target tissue (74). Suppressing endocytosis in macrophages by knocking down clathrin, a key protein involved in endocytosis (75), via an EV delivery strategy, improved cardiac targeting of therapeutic EVs containing miR-21a-5p and decreased the cardiotoxic effects of subsequently applied DOX (74). Combining cardiac-targeting peptides with biomaterials can further improve retention of therapeutic EVs in cardiac tissue. For example, encapsulating EVs isolated from human umbilical cord MSCs in a hydrogel containing cardioprotective peptides improved retention and cardiac function post MI in rats (76). In addition, conjugating cardiac stem cell-derived EVs with a homing peptide sequence greatly enhanced their protective ability, targeting them more specifically to the heart (77, 78). Precision targeting of EVs within the body is another critical component of successfully implementing preventative molecular strategies.

## Discussion

**Figure 2:**
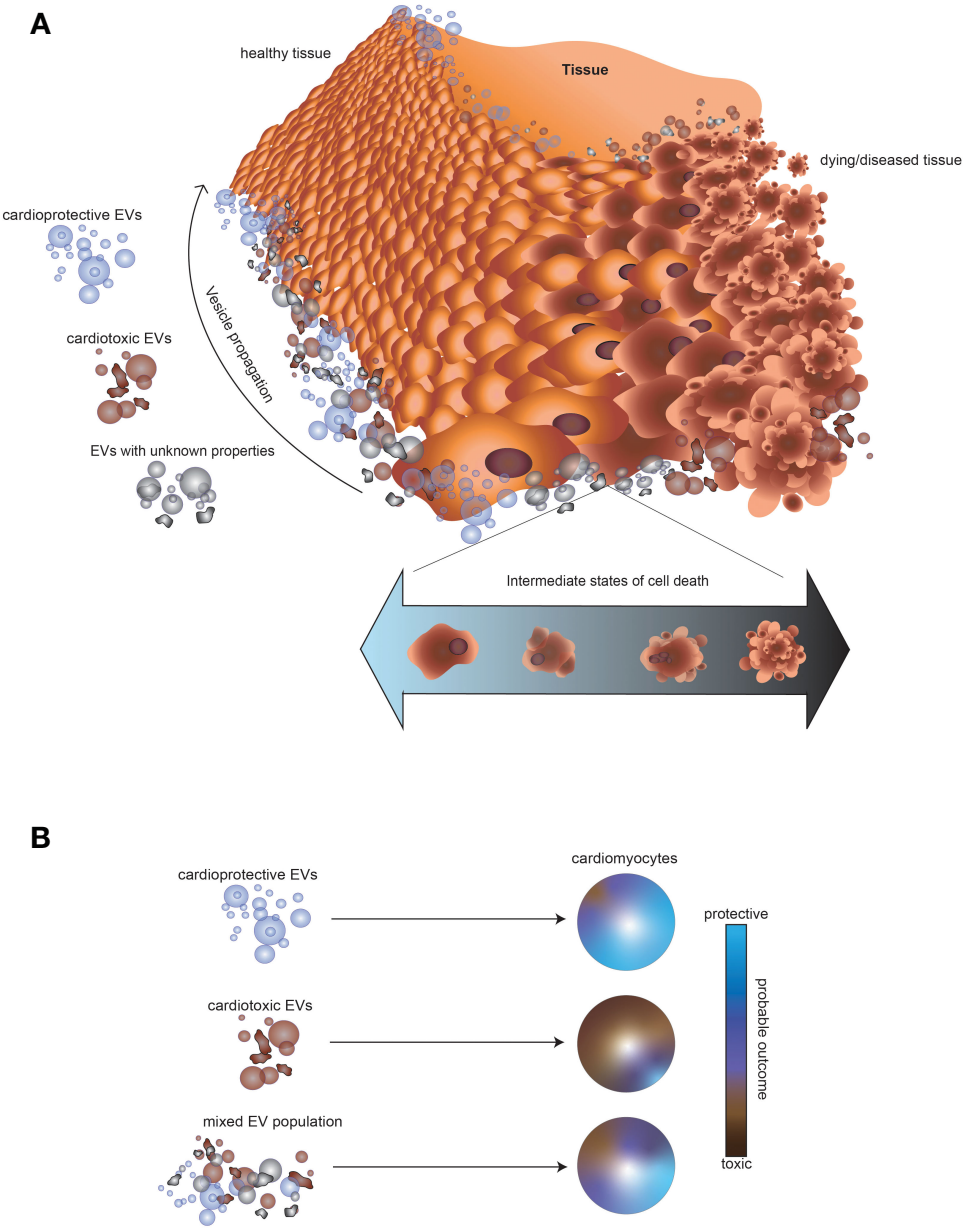
Impact of mixtures of EVs released by cells in different states of growth and cellular death on the surrounding tissue. **(A)** Illustration of the possible outcome of a toxic exposure on EV release in cardiac tissue. A transient toxic exposure in one part of the tissue could create a mixture of cell populations: dying cells, unaffected cells, and cells recovering from a death pathway. Consequently, the surrounding tissue would be expected to encounter a mixture of EVs, consisting of vesicles with cardioprotective, cardiotoxic, or unknown signaling potential. **(B)** Schematic illustrating that EVs from some cell types might result in a higher probability of a cardioprotective outcome (blue), whereas other EVs might result in a cardiotoxic outcome (brown). However, the impact of a mixture of EVs with either cardioprotective or cardiotoxic potential is difficult to predict.

Current strategies for monitoring chemotherapy patients for cardiotoxicity include echocardiography and molecular biomarkers, most commonly c-troponin and N-terminal-pro-brain natriuretic peptide. However, these assessments may fail to identify the earliest stages of cardiotoxicity, prior to damage taking place (79). Novel biomarkers, such as EVs, are needed toward detection of impending deleterious events. A deeper understanding of EV makeup in response to specific cellular events within a tissue is critical not just for diagnostic purposes, but also for understanding how the balance of EVs impacts surrounding cells and overall tissue health. Since tissues are comprised of multiple cell populations undergoing heterogeneous events, EV tissue-specific signatures are likely complex mixtures of vesicles with diverse cargo and properties. For example, a transient toxic exposure triggering death of cells in one part of a tissue, and subsequent recovery of some cells might be expected to generate a mixture of EVs with cardiotoxic or unknown signaling potential (**Figure 2A**). The surrounding healthy cardiac tissue would thus be subjected to a mixture of EVs with cardioprotective, cardiotoxic, or unclear cardiac signaling potential. The balance of cardioprotective and cardiotoxic EVs in a tissue would be expected to determine the probable fate of the surrounding cells, but may be increasingly hard to predict in complex EV mixtures (**Figure 2B**). Further research is needed to better understand EV release in response to transient application of cardiotoxins and recovery from cell death pathways.

Predicting the probable outcome of exposing healthy cardiac tissue to a mixture of EVs is made yet more difficult by the complex relationship between cellular states. For example, cell death is not always a sign of toxicity or poor tissue health. Remarkably, the dependence of tissue repair processes on cell death, termed “regenerative cell death” has been observed in several species and contexts (80, 81). In these studies, EVs from apoptotic cells did not appear to play a toxic role. Rather, the detected apoptotic bodies were engulfed by other neighboring cells (82). However, the extent to which EVs were involved directly in these regenerative processes was not fully explored.

An exciting prospect would be if specific EV signatures could be mapped to individual pathways of toxicity or demise, enabling personalized therapeutic strategies and precision prevention of cardiotoxicity. Toward this end, a better understanding of what types of EVs are released from cells undergoing specific cell death pathways and cells in intermediate stages of cell death would be valuable. In addition, off-target cardiotoxic effects of anticancer drugs can potentially be minimized through encapsulation or parallel administration with cardioprotective EVs. However, to fully develop these strategies, a thorough understanding of the factors that contribute to producing EVs with cardioprotective properties is critical. Rather than focusing on simple addition of cardioprotective EVs, a personalized medicine approach balancing the proportions of multiple EV types may increase the probability of a cardioprotective or regenerative outcome.

## Data availability statement

The original contributions presented in the study are included in the article/supplementary material. Further inquiries can be directed to the corresponding authors.

## Author contributions

BS: Conceptualization, Formal analysis, Methodology, Project administration, Resources, Supervision, Visualization, Writing – original draft, Writing – review & editing. AK: Writing – original draft, Writing – review & editing. MW: Conceptualization, Writing – original draft, Writing – review & editing. KG: Conceptualization, Funding acquisition, Resources, Supervision, Writing – original draft, Writing – review & editing. ET: Conceptualization, Funding acquisition, Resources, Supervision, Writing – original draft, Writing – review & editing.
